# High resolution crystal structure of PedB: a structural basis for the classification of pediocin-like immunity proteins

**DOI:** 10.1186/1472-6807-7-35

**Published:** 2007-05-30

**Authors:** In-Kwon Kim, Min-Kyu Kim, Ji-Hye Kim, Hyung-Soon Yim, Sun-Shin Cha, Sa-Ouk Kang

**Affiliations:** 1 Laboratory of Biophysics, School of Biological Sciences, and Institute of Microbiology, Seoul National University, Seoul 151-742, Republic of Korea; 2 Beamline Division, Pohang Accelerator Laboratory, Pohang 790-784, Republic of Korea

## Abstract

**Background:**

Pediocin-like bacteriocins, ribosomally-synthesized antimicrobial peptides, are generally coexpressed with cognate immunity proteins in order to protect the bacteriocin-producer from its own bacteriocin. As a step for understanding the mode of action of immunity proteins, we determined the crystal structure of PedB, a pediocin-like immunity protein conferring immunity to pediocin PP-1.

**Results:**

The 1.6 Å crystal structure of PedB reveals that PedB consists of an antiparallel four-helix bundle with a flexible C-terminal end. PedB shows structural similarity to an immunity protein against enterocin A (EntA-im) but some disparity to an immunity protein against carnobacteriocin B2 (ImB2) in both the C-terminal conformation and the local structure constructed by α3, α4, and their connecting loop. Structure-inspired mutational studies reveal that deletion of the last seven residues of the C-terminus of PedB almost abolished its immunity activity.

**Conclusion:**

The fact that PedB, EntA-im, and ImB2 share a four-helix bundle structure strongly suggests the structural conservation of this motif in the pediocin-like immunity proteins. The significant difference in the core structure and the C-terminal conformation provides a structural basis for the classification of pediocin-like immunity proteins. Our mutational study using C-terminal-shortened PedBs and the investigation of primary sequence of the C-terminal region, propose that several polar or charged residues in the extreme C-terminus of PedB which is crucial for the immunity are involved in the specific recognition of pediocin PP-1.

## Background

Bacteriocins are ribosomally-synthesized antimicrobial peptides produced by bacteria. Most bacteriocins are generally synthesized as prepeptides and can be classified into several classes depending on their post-translational processing and the mode of action [1-3]. Type I bacteriocins, of which a nisin is most well-known, undergo post-translational modifications such as formation of dehydrated residues and lanthionine bridges [4]. In contrast, type IIa bacteriocins are matured by simple cleavage of a leader peptide and characterized by a conserved YGNGVXC motif in the N-terminus [5-7]. These bacteriocins contain 37~48 residues and show potent activity against related Gram-positive bacteria, e.g. *Listeria *spp. [8,9]. NMR studies revealed that type IIa bacteriocins consists of two domains, a cationic N-terminal domain and a hydrophobic C-terminal domain [10-12]: the N-terminal domain interacts with the anionic cell surface of Gram-positive bacteria [13,14], while the C-terminal domain participates in the membrane permeabilization [7,15,16].

In general, type IIa bacteriocins are coexpressed with cognate immunity proteins (pediocin-like immunity proteins) composed of 88~115 amino acids, in order to protect the organism from the antimicrobial activity of its own bacteriocin [17-21]. It is known that the immunity proteins usually exhibit high specificity against their cognate bacteriocins. In contrast to the type IIa bacteriocins that exhibit high sequence conservation in their N-terminus, the degree of sequence homology between the pediocin-like immunity proteins varies (5~85%), which provides the basis for classification of them into three subgroups (A, B, and C) [22,23]. The mode of action of immunity proteins and the basis of their specificity is still unclear, although the C-terminal half of immunity proteins is known as an important determinant for the specific recognition of their cognate bacteriocins [24,25]. However, because pediocin-like immunity proteins don't interact directly with their cognate bacteriocins [6,20,26] and exist in cytoplasm [18,20], it has been proposed that they may act by interfering with the formation of a functional pore complex in the membrane or blocking the pore itself. The accumulated structural information for certain class of immunity proteins could generally provide an important clue to their mode of action. However, although a number of pediocin-like immunity proteins have been identified and characterized, so far, only two structures have been reported: immunity proteins conferring immunity to enterocin A (EntA-im) [27] which belongs to subgroup A, and carnobacteriocin B2 (ImB2) [28] which belongs to subgroup C.

Pediocin secreted by *Pediococcus *spp. is a representative of the type IIa bacteriocins. Among pediocins, pediocin PA-1 and its operon of *Pediococcus acidilactici *PAC1.0 is the most well known [26,29]. Recently, we identified another pediocin (pediocin PP-1) operon (*pedA*, *pedB*, *pedC*, and *pedD*) from *Pediococcus pentosaceus *CBT-8, which is isolated from the Korean traditional fermentative food, kimchi. Based on the remarkable degree of sequence identity (~99%) of PedB of *P. pentosaceus *to that of *P. acidilactici*, we regarded PedB as the immunity protein against pediocin PP-1. PedB is a small positively charged protein with 112 amino acids and belongs to subgroup A. No transmembrane helices are predicted from sequence analysis. To provide a structural insight into the function of PedB, we determined the three dimensional structure of PedB at 1.6 Å resolution and suggested the basis for the structural classification of pediocin-like immunity proteins, and examined the role of the C-terminal end of PedB.

## Results and discussion

### PedB confers immunity to pediocin PP-1

**Figure 1:**
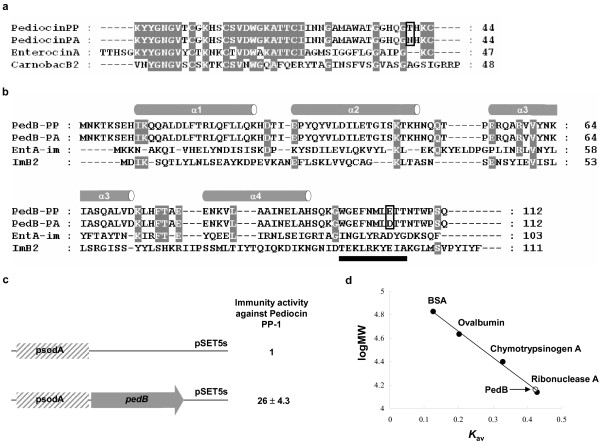
(A) Sequence alignment of pediocin PP-1 from *P. pentosaceus *(pediocinPP) with pediocin PA-1 from *P. acidilactici *(pediocinPA), EnterocinA, and Carnobacteriocin B2 (CarnobacB2). The substitution between pediocin PP-1 and pediocin PA-1 is indicated by black box. The color scheme of white on dark grey indicates the consensus residue derived from the occurrence of >70% of a single residue at a given position. (B) Structure-based alignment of PedB from *P. pentosaceus *(PedB-PP) with PedB from *P. acidilactici *(PedB-PA), EntA-im, and ImB2. Secondary structure of PedB is presented above the alignment. The additional fifth helix of ImB2 is indicated by black bar. The color scheme is same to (A). The conservative substitution between PedB proteins for pediocin PP-1 and pediocin PA-1 is indicated by black box. (C) Pediocin PP-1 susceptibility of *L. sakei *strain harboring *pedB *gene and control plasmid. The MIC is the concentration of bacteriocin that inhibited growth of the indicator strain by 50%. The immunity activity is presented as the -fold increase in MIC observed for strains expressing PedB variants relative to MICs for strains containing only the control plasmid. The results represent the averaged data from at least three experiments. The psodA indicates the promoter of *sodA *encoding Mn-containing superoxide dismutase in *B. subtilis*. (D) Determination of oligomeric state of PedB. Oligomeric state of PedB was analysed by gel filtration chromatography. Predicted molecular mass of PedB (See Methods) is 14,428 Da (*K*_av _= 0.42), indicating that PedB exists as a monomer in solution.

We assumed PedB as an immunity protein to pediocin PP-1 on the basis of sequence identity to that of *P. acidilactici *(only one conservative substitution (Glu for Asp) at a position of 104 exists between two PedB proteins for pediocin PP-1 and pediocin PA-1) (Figure 1b). To investigate whether PedB confers immunity to pediocin PP-1, we introduced the *pedB *gene into the bacteriocin-sensitive *Lactobacillus sakei *NCFB 2714 strain [24] and tested the susceptibility against ammonium sulphate-precipitated fermentate of *P. pentosaceus *containing pediocin PP-1 (See Methods). Because the *pedB *gene doesn't have its own promoter, we used the promoter of *sodA *encoding Mn-containing superoxide dismutase in *Bacillus subtilis *[30,31]. *L. sakei *harboring the *pedB *gene was about 26 times less susceptible compared to the strain possessing only the *sodA *promoter, indicating that PedB successfully acted as the immunity protein to pediocin PP-1 (Figures 1c &4). However, when PedB purified from *E. coli *was mixed with pediocin PP-l solution, PedB failed to inactivate the bacteriocin (see Additional file 1), which is in line with the previous report that pediocin-like immunity proteins don't directly interact with their cognate bacteriocins [6,20,26].

### Overall structure of PedB

The native PedB and selenomethionine-substituted PedB mutant (L24M) (See Methods) were crystallized under similar conditions. The initial model was built according to the phased electron density map obtained by using anomalous scattering differences of selenium atoms in the selenomethionyl L24M crystal. A final model for native PedB was refined subsequently to 1.6 Å resolution against native data. The native PedB crystals contain one molecule in the asymmetric unit. Out of the total 112 amino acids in native PedB, residues 7–93 are visible in the crystal structure. No significant structural difference was observed between the native and mutant PedB (r.m.s.d of 0.3 Å for all C_α _atoms).

**Figure 2:**
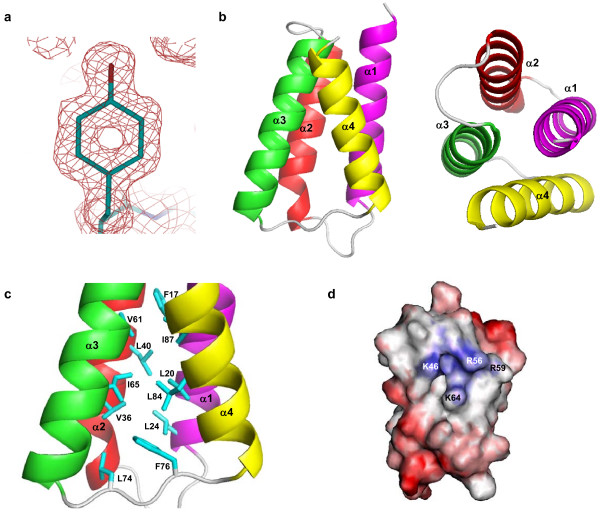
(A) A 2*F*_o_-*F*_c _electron density map of the Tyr62 residue contoured at 1σ. The hole in the map indicates the high quality and resolution of the electron density map. (B) A ribbon diagram showing the overall structure (left) and the top view (right) of PedB shown with secondary structures labeled. (C) A ribbon diagram showing the hydrophobic core of PedB with secondary structures and important residues labled. (D) Surface charge representation of PedB on the view of α2 and α3. Blue and red colors indicate positive and negative charge, respectively.

The crystal structure reveals that PedB forms a compact globular domain composed of four helices (Figure 2b). They are arranged so that α1 and α3 run in the same direction and α2 and α4 in the opposite direction, forming an antiparallel four-helix bundle (Figure 2b). The relative disposition of the four helices of PedB is maintained mainly by a network of hydrophobic interactions in the core of the molecule: Phe17, Leu20, and Leu24 from α1 (residues 9–26), Leu48 from α2 (residues 31–49), Val61 and Ile65 from α3 (residues 55–72), Leu74 and Phe76 from the loop between α3 and α4, Leu84 and Ile87 from α4 (residues 80–92) form a hydrophobic core (Figure 2c). This structure is further stabilized by several polar interactions between helices and between helix and loop: the side-chain of Gln21 makes polar contact with Glu41, Tyr35 with Gln68, Gln52 with Gln57, Tyr62 with Asn88, His27 with Tyr33 and Glu80 (figure not shown). The antiparallel helices are linked by a short loop of four to seven residues between them. The C-terminal end of PedB (residues 94–112) that is thought to be important for immunity has no electron density, suggesting that this region is flexible. We could not find any oligomeric contacts between PedB monomers in crystal packing, which is consistent with the result that PedB exists as a monomer in solution (Figure 1d).

### Structural comparison of PedB to EntA-im and ImB2

Until now, two structures for pediocin-like immunity proteins, the crystal structure of EntA-im [27] and the solution structure of ImB2 [28], have been determined. Although PedB exhibits a limited sequence identity to EntA-im (14%) and ImB2 (6%) (Figure 1b), these three immunity proteins share an antiparallel left twisted four-helix bundle as a conserved scaffold. In addition, a positively charged region whose physiological function is obscure but which is commonly found in EntA-im, ImB2, and even in the structure models of pediocin-like immunity proteins [27,28], is also observed in the surface representation of PedB. In PedB, the positively charged region is formed by residues from α2 (Lys46) and α3 (Arg56, Arg59 and Lys 64) (Figure 2d).

**Figure 3:**
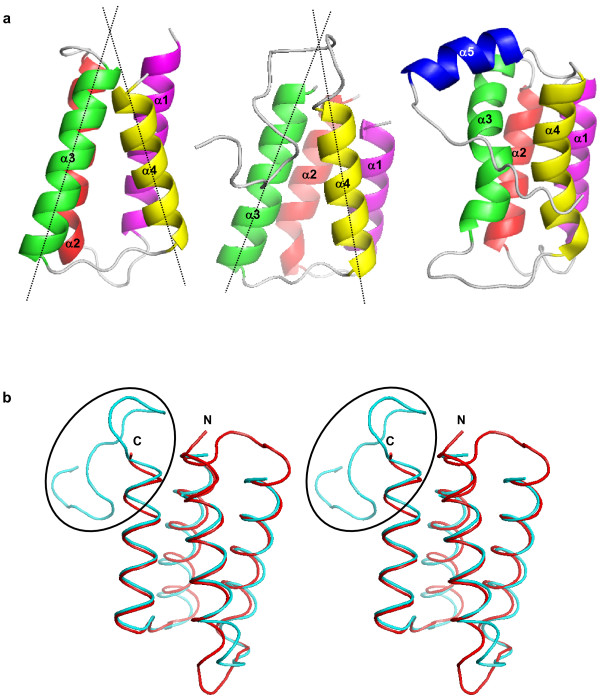
(A) Ribbon diagrams of PedB (left), EntA-im (middle), and ImB2 (right) shown with secondary structures labeled. The axes of α3 and α4 are indicated by dotted lines. The angle between α3 and α4 is 33° for PedB and 31° for EntA-im. (B) Stereo view of the superimposed structures of PedB (red) and EntA-im (cyan). The flexible C-terminal loop of EntA-im that is thought to be important for its immunity is highlighted by a black ellipse. N and C indicate the N- and C-terminus of PedB, respectively.

Pediocin-like immunity proteins are divided into three subgroups on the basis of the sequence homology [23,28]. PedB and EntA-im belong to the subgroup A, and ImB2 belongs to the subgroup C. The fact that three immunity proteins share a common scaffold but are classified into different subgroups, aroused an interest in whether each subgroup has a conserved structural fingerprint. To test this, we compared the structure of PedB with those of EntA-im and ImB2, respectively. As expected, the structure of PedB is well-superimposed to that of EntA-im with r.m.s.d. of 1.3 Å for all C_α _atoms (Figure 3b). They show a good agreement in the interhelical angles and length of helices. The structural similarity between PedB and EntA-im is much higher than expected from the degree of sequence identity [32].

**Figure 4:**
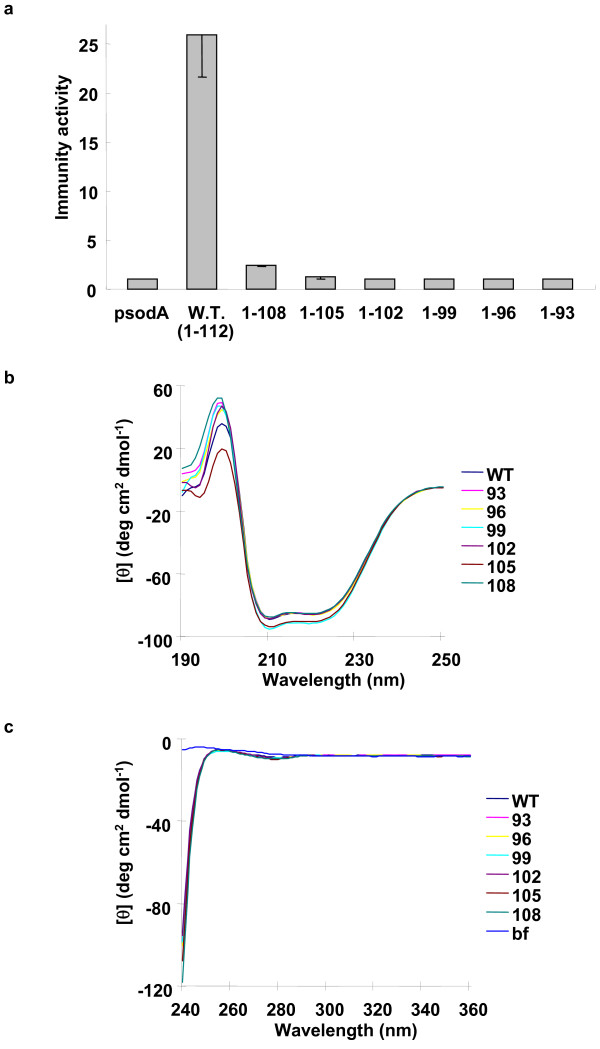
(A) A graphic display of the immunity activity of *L. sakei *strains possessing PedB and PedB variants to pediocin PP-1. The results are presented using the same methods as in Figure 1c. The results represent the averaged data from at least three experiments, and the standard deviation values are indicated in each bar. (B) Far-UV CD spectra of PedB and C-terminal shortened PedB variants. CD spectra (190~250 nm) of 25 μg/ml ml wild type PedB and PedB variants were obtained to compare the secondary structure at 25°C. (C) Near-UV CD spectra of PedB and C-terminal shortened PedB variants. CD spectra (240~360 nm) of 200 μg/ml wild type PedB and PedB variants were obtained to compare the tertiary structure and protein folding at 25°C.

In contrast, compared with PedB and EntA-im, ImB2 shows a considerable disparity in both the C-terminal end and the core structure. Consistently, the r.m.s.d. value of 2.8 Å between PedB and ImB2 is higher than that of 1.3 Å between PedB and EntA-im. ImB2 contains an additional helix at the C-terminus, whereas the C-termini of PedB and EntA-im are flexible. In addition, the starting region (residues 31–33) of the second helix of PedB and EntA-im forms a 3_10 _helix, which is not found in ImB2 (Figures 3a). The most remarkable structural difference in the four-helix bundle scaffold between PedB/EntA-im (subgroup A) and ImB2 (subgroup C) is observed in the local structure constructed by α3, α4, and a connecting loop. In PedB and EntA-im, α3, α4, and a straightened loop between them form a triangle-like conformation with an interhelical angle of ~30° (Figure 3a). Two hydrophobic residues from the connecting loop (Leu74 and Phe76 of PedB, and Ile67 and Phe69 of EntA-im) penetrate into the interhelical space between α3 and α4, participating in hydrophobic core formation (Figures 2c & 3a). As a result, the triangle-like conformation made by α3, α4, and the loop between them is stabilized. However, in ImB2, the connecting loop between α3 and α4 is not implicated in hydrophobic core formation. Instead the loop is located outside the four helix bundle structure. Consequently, α3 and α4 of ImB2 is arranged in a nearly parallel manner, extensively contacting with each other along the helical axis (Figure 3a). In conclusion, in spite of the overall similarity in structure, the difference of the C-terminal conformation and the local structure constructed by α3, α4, and the connecting loop between them can be used for the classification of pediocin-like immunity proteins.

### Extreme C-terminal end of PedB is essential for immunity

The fact that PedB and EntA-im have almost identical four-helix bundle structures but their cognate bacteriocins are not identical (Figure 1a), led us to hypothesize that the flexible C-terminus region of PedB is critical for specific recognition of pediocin PP-1. Actually, according to the sequence alignment between PedB and EntA-im (Figure 1b), the sequence conservation of this region is much lower than that of other region. In order to verify the role of the C-terminal end, we introduced several shortened PedBs (PedB_1–93_, PedB_1–96_, PedB_1–99_, PedB_1–102_, PedB_1–105_, and PedB_1–108_) into *L. sakei *and tested the susceptibility to pediocin PP-1.

As shown in Figure 4a, deletion of last four residues (PedB_1–108_) resulted in severe loss of immunity compared to the wild type PedB, and deletion of seven residues (PedB_1–105_) almost completely abolished the activity. Since the flexible C-terminal end is not involved in the folding of PedB, the deletion cannot disturb the core structure of PedB. Actually, in the far-UV spectral region (190~250 nm), the CD spectra of PedB variants were highly similar to that of the wild type PedB (Figure 4b), which exhibits two negative bands at 208 and 222 nm supporting the all alpha helical structure of PedB [33]. Furthermore, in the near-UV spectral region (240~360 nm), PedB variants showed almost identical CD spectra to that of the wild type PedB (Figure 4c), indicating that PedB and PedB variants have very similar tertiary structure. Therefore, the mutant study indicates that the extreme C-terminus is crucial for the PedB immunity. Interestingly, in the flexible C-terminal end, several polar or charged residues are located from the position of 104 (Glu104, Thr105, Thr106, Asn107, Thr108, Ser111, and Gln112). The significant loss of immunity on the deletion of extreme C-terminus suggests that polar interactions mediated by these polar or charged residues might be involved in the specific recognition of pediocin PP-1.

## Conclusion

We have determined the 1.6 Å crystal structure of PedB. The three pediocin-like immunity proteins whose structures are known (subgroup A and C) share a four helix bundle structure. In addition, the subgroup B immunity proteins, such as sakacin P and piscicolin 126 [23], are also predicted to consist of four helices by secondary structure prediction using the Jpred server [34] (data not shown), strongly suggesting that the four helix bundle is a conserved scaffold in the pediocin-like immunity proteins. The structural comparison among pediocin-like immunity proteins reveals that the C-terminal conformation and the local structure constructed by α3, α4, and the connecting loop between them exhibits a considerable disparity according to subgroup type, indicating that the pediocin-like immunity proteins could be classified into subgroups based on the structure as well as the sequence homology. The mode of action of pediocin-like immunity proteins still remains elusive. However, our results demonstrate that the extreme C-terminus of PedB with several charged residues is essential for immunity. Further analysis about what is the membrane-bound receptor for type IIa bacteriocins and how the recognition between the immunity proteins and cognate bacteriocins is achieved in a highly specific manner, is a great challenge in the future.

## Methods

### Bacterial strains and growth conditions

Pediocin PP-1 was produced by *P. pentosaceus *CBT8. *L. sakei *NCFB 2714 containing a plasmid with or without inserted PedB variants was used as an indicator strain for assaying immunity activity against pediocin PP-1. For overexpression and selenomethionine-labelling, *E. coli *strain BL21 (DE3) and B834 (DE3) (Novagen) were used respectively. *P. pentosaceus *and *L. sakei *were grown in MRS broth (Difco) at 30°C, and *E. coli *strains were grown in Luria-Bertani medium at 37°C. Chloramphenicol (Sigma) was used at 5 μg ml^-1 ^for *L. sakei *and 34 μg ml^-1 ^for *E. coli*. Ampicillin (Sigma) was used at 100 μg ml^-1^.

### Construction of plasmids expressing PedB variants and transformation

In order to construct a plasmid expressing the wild type *pedB *gene under the control of the promoter of *sodA*, DNA fragments containing the *pedB *ORF were amplified by polymerase chain reaction (PCR) using two primers possessing *Nde*I and *Bam*HI site, respectively, and cloned into pGEMTeasy (Promega) generating pGEMT-pedB. *Eco*RI-*Bam*HI fragment of pGEMT-pedB was ligated into pGEX4T-1, generating pGEX-pedB. The *sodA *promoter was amplified by PCR using *B. subtilis *genomic DNA as a template and cloned into pGEMTeasy, generating pGEMT-psodA. This plasmid was digested with *Sal*I-*Nde*I and inserted into the pGEX-pedB, generating pGEX-psodA:pedB. *Sal*I-*Bam*HI fragment of this plasmid was ligated into pSET5s [35], generating pSET-psodA:pedB. As a control, *Sal*I-*Eco*RI fragment of pGEMT-psodA was also cloned to pSET5s, generating pSET-psodA. To construct plasmids for shortened PedB variants, each PedB variant was amplified by PCR with proper primer pairs containing stop codon at positions of 94, 97, 100, 103, 106, and 109, respectively. And then, 0.35 kb *Nde*I-*Bam*HI fragments of the PCR products were exchanged with *pedB *in pGEX-psodA:pedB. Finally, *Sal*I-*Bam*HI fragment of these plasmids were moved to pSET5s.

The expression constructs were electroporated into the *L. sakei *using Bio-Rad gene pulser as described by Aukrust *et al*. [36]. Briefly, *L. sakei *was made electrocompetent as follows. Cells were grown up to OD_600 _of approximately 0.5 in MRS broth with 2% (w/v) glycine, and then were washed twice with 1 mM MgCl_2 _followed by washing twice with 30% (w/v) polyethylene glycol 1500.

### Protein purification, crystallization, and structure determination

PedB was expressed in *E. coli *and purified as described previously [37]. To ensure an anomalous signal in the X-ray diffraction experiment one leucine (Leu24) was mutated to methionine (L24M). For the selenomethionine labeling of PedB-L24M mutant, methionine auxotroph *E. coli *B834 (DE3) (Novagen) strain was used as a host for plasmid transformation. The selenomethionyl PedB-L24M protein was purified by an identical procedure to that for the wild-type PedB. The purified SeMet PedB-L24M was concentrated to approximately 10 mg ml^-1 ^for crystallization.

Native PedB crystals (*P*6_2_; *a *= *b *= 62.2 and *c *= 39.9 Å, a monomer in asymmetric unit) were grown and a 1.6 Å data set was obtained with a Bruker Proteum 300 CCD at Beamline 6B at Pohang Light Source as described previously [37].

The selenomethionyl PedB-L24M crystals were produced under the condition containing 2.4 M ammonium sulfate, 0.3 M sodium chloride, and 0.1 M MES (pH 6.0). These crystals belonged to space group *P*6_2 _with cell parameters *a *= *b *= 62.6, and *c *= 39.8 Å, and contained one monomersper asymmetric unit. Diffraction data were processed and scaled with programs *DENZO *and *SCALEPACK *from the *HKL *program suite [38]. The structure was solved by the multiwavelength anomalous dispersion (MAD) method using three-wavelength data sets of the selenomethionyl L24M mutant crystal: the peak wavelength was at 0.97900 Å, and the inflection wavelength was at 0.97911 Å, and the remote wavelength was at 0.97137 Å. Two selenium sites were located, and phase refinement was done by using the programs *SOLVE *and *RESOLVE *[39,40]. Initial phasing and model building was done with L24M MAD data set. Model building was done using the program *QUANTA *software (Molecular Simulations) and refined with *CNS *[41] to an *R*_free _of 0.276 and *R*_work _of 0.256 in the resolution range of 20-1.9 Å. The native PedB structure was solved by molecular replacement with CNS using the L24M structure as a search model, and refined to *R*_free _of 0.225 and *R*_work _of 0.192 in the resolution range of 20-1.6 Å. The refinement statistics for the native PedB structure are summarized in Table 1. The ideality of the model stereochemistry was verified by *PROCHECK *[42]. The Ramachandran plots indicate that 96.3% of non-glycine residues are in the most favored regions, and 2.4% in the additionally allowed regions, and 1.2% in the generously allowed regions. Coordinate has been deposited with the Protein Data Bank under the accession code 2IP6.

**Table 1:** Summary of crystallographic analysis.

*Data collection*				
Data set	L24M selenium data			Native PedB
Space group	*P*6_2_			*P*6_2_
Unit-cell parameters (Å)	a = b = 62.6 Å, c = 39.8 Å			a = b = 62.2 Å, c = 39.9 Å
Wavelength (Å)	0.97900	0.97911	0.97137	0.91841
Resolution (Å)	50-1.9	50-1.9	50-1.9	30-1.6
Completeness (%)^a^	99.4 (98.0)	99.4 (97.5)	99.2 (97.7)	98.4 (97.1)
*R*_sym _(%)^a,b^	9.9 (32.0)	9.9 (34.4)	10.2 (52.4)	6.0 (19.4)
Average I/σ	20.2 (3.0)	19.1 (2.7)	17.5 (4.0)	79.8 (15.8)

*Refinement statistics*				

Resolution range (Å)				30-1.6
Number of reflections	7,087	7,090	7,072	11,539
Total number of atoms				
Total				868
Water				186
Completeness of data (%)				98.4
*R*^c ^(*R*_free_) (%)				19.2 (22.5)
r.m.s. deviations^d^				
Bonds (Å)				0.004
Angles (°)				0.945

**a. **The number in parentheses is for the outer shell.

**b**. *R*_sym _= Σ_h_Σ_I_|I_h,i _- I_h_|/Σ_h_Σ_I_I_h,i_, where I_h _is the mean intensity of the i observation of symmetry related reflections of h.

**c. ***R *= Σ|*F*_o_-*F*_c_|/Σ*F*_o_, where *F*_o _= *F*_p_, and *F*_c _is the calculated protein structure factor from the atomic model. *R*_free _was calculated with 10% of the reflections.

**d. **Root mean square (r.m.s.) deviations in bond length and angles are the deviations from ideal values.

### Determination of oligomeric state of PedB

Purified recombinant PedB and low molecular mass standards (Amersham Biosciences) were applied on a Superdex 75 16/60 column (Amersham Biosciences) preequilibrated with 20 mM Tris-HCl (pH 8.0) containing 150 mM NaCl. A standard curve was generated by plotting the logarithm of molecular mass of standard proteins against their *K*_av_, where *K*_av _= (*V*_e _- *V*_o_)/(*V*_t _- *V*_o_): *V*_e_, elution volume; *V*_o_, void volume; *V*_t_, total bed volume. *K*_av _of PedB determined by using the same column was compared to the profile of protein standards: bovine serum albumin (67 kDa), ovalbumin (43 kDa), chymotrypsinogen A (25 kDa), and ribonuclease A (13.7 kDa).

### Purification of C-terminal shortened PedB mutants in E. coli

To construct plasmids for expression of shortened PedB variants in *E. coli*, each PedB variant was amplified by PCR with proper primer pairs containing stop codon at positions of 94, 97, 100, 103, 106, and 109, respectively. And then, 0.35 kb *Bam*HI-*Sal*I fragments of the PCR products were cloned into pGEX-4T-1. Each PedB variant was expressed and purified by an identical procedure to that for the wild type PedB.

### CD Spectroscopy

All of the CD experiments were performed on a Jasco J-715 spectropolarimeter using a 0.2 cm path-length cell, with a 1 nm bandwidth and 4 s response time. Far- and near-UV CD spectra were collected from 250 to 190 nm and from 360 to 240 nm, respectively, with a scan speed of 100 nm/min and 1 nm step resolution. Three individual scans were added and averaged. A buffer containing 20 mM Tris-HCl (pH 7.4) and 150 mM NaCl was used for CD spectra.

### Bacteriocin assay

For the bacteriocin assay, we used an ammonium sulphate-precipitated fermentate of *P. pentosaceus *containing pediocin PP-1. After the ammonium sulphate was added to the culture supernatant of *P. pentosaceus *to 80% saturation, the precipitate was dissolved in 10 mM sodium acetate pH 5.0. When this ammonium sulphate-precipitated fermentate was applied to the spot test against lawns of *Listeria monocytogenes *and *Listeria innocua *on agar media, it showed strong inhibitory effect (see Additional file 1). However, when proteinase K and chymotrypsin were treated to the fermentate, the clear zone disappeared (data not shown), indicating that the growth inhibition was due to pediocin PP-1 peptide.

Bacteriocin activity was measured using a microtiter plate assay system as described by Nissen-Meyer *et al*. [43]. Briefly, each well of a plate contained 100 μl of MRS medium mixed with ammonium sulphate-precipitated fermentate of *P. pentosaceus *at 1.5 fold dilution and *L. sakei *strain that is 1000 fold diluted at an OD_600 _of 1. This plate was incubated overnight at 30°C, and then the growth was measured at 595 nm using a microplate reader. The minimal inhibitory concentration (MIC) was defined as the concentration of bacteriocin that inhibited growth of the indicator strain by 50%. The MICs presented here are the results of three to four independent measurements. Transformed indicator strains were grown in the presence of 5 μg ml^-1 ^chloramphenicol to maintain the plasmids in the cells. The immunity activity of PedB was calculated by comparing MIC values for a strain expressing the PedB variants with those for the strain possessing only the *sodA *promoter.

## Authors' contributions

IK carried out the protein expression, purification, and crystallization experiments, participated in structure determination, construction of PedB-expressing plasmids, and bacteriocin assay, and wrote the initial version of the manuscript. MK carried out the site-directed mutagenesis, mutant purification, crystallization and participated in data analysis, structure determination, and electroporation. JK participated in the electroporation and bacteriocin assay. HY participated in data analysis and design of this study. SC and SK conceived the project, supervised the project, participated in its design and coordination, and helped to draft the manuscript. All authors read and approved the final manuscript.

## Supplementary Material

Additional file 1 **Detection of pediocin PP-1 activity in the absence and presence of PedB. **The ammonium sulphate-precipitated fermentate was applied to the spot test against lawns of *L. innocua *and *L. monocytogenes *on agar media. Click here for file
