# Supplementary figures and images for: High resolution crystal structure of PedB: a structural basis for the classification of pediocin-like immunity proteins
Source: BMC Struct Biol. 2007 May 30;7:35. doi: 10.1186/1472-6807-7-35 (PMC1904221; doi:10.1186/1472-6807-7-35)

**Pediocin PP-1**

**Pediocin PP-1  
+PedB**

***L. innocua***

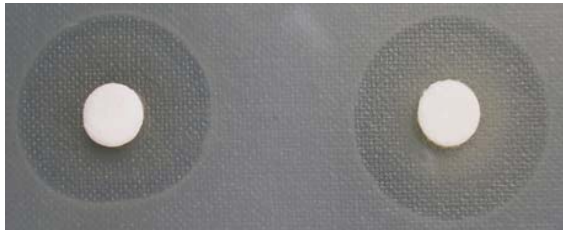

***L. monocytogenes***

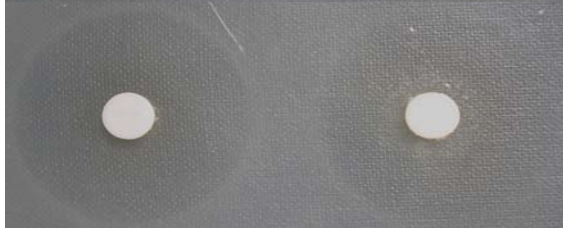

Supplement: Additional file 1 — Detection of pediocin PP-1 activity in the absence and presence of PedB. The ammonium sulphate-precipitated fermentate was applied to the spot test against lawns of L. innocua and L. monocytogenes on agar media. [file 1472-6807-7-35-S1.pdf]
